# Examining Relationships between Metabolism and Persistent Inflammation in HIV Patients on Antiretroviral Therapy

**DOI:** 10.1155/2018/6238978

**Published:** 2018-09-27

**Authors:** Duale Ahmed, David Roy, Edana Cassol

**Affiliations:** ^1^Department of Biology, Carleton University, Ottawa, Ontario, Canada; ^2^Department of Health Sciences, Carleton University, Ottawa, Ontario, Canada

## Abstract

With the advent of antiretroviral therapy (ART), HIV-infected individuals are now living longer and healthier lives. However, ART does not completely restore health and treated individuals are experiencing increased rates of noncommunicable diseases such as dyslipidemia, insulin resistance, type 2 diabetes, cardiovascular disease, and nonalcoholic fatty liver disease. While it is well known that persistent immune activation and inflammation contribute to the development of these comorbid diseases, the mechanisms underlying this chronic activation remain incompletely understood. In this review, we will discuss emerging evidence that suggests that alterations in cellular metabolism may play a central role in driving this immune dysfunction in HIV patients on ART.

## 1. Introduction

HIV infection is now considered a chronic disease requiring lifelong treatment with combination ART. Infected individuals are living longer, healthier lives with near normal life expectancies [1, 2]. However, ART does not completely restore health and treated individuals are experiencing increased rates of non-AIDS-associated comorbidities such as cardiovascular disease (CVD), type 2 diabetes, neurocognitive impairment, and cancer [3–5]. These comorbid diseases represent a significant problem for the long-term management of HIV patients, particularly as recent studies suggest that screening algorithms and treatments may not be as effective in infected populations [6–9]. By 2020, it is expected that >30 million people living with HIV will have access to ART [10]. Progress towards improving outcomes for these individuals will depend on the identification of novel strategies for the prevention and treatment of these non-AIDS- associated comorbities.

Chronic immune activation and inflammation persist in HIV patients on ART [11–14]. This immune dysfunction is associated with hypercoagulation, tissue fibrosis/damage, and organ system dysfunction, which over time contribute to the development of non-AIDS-associated comorbidities [15–17]. The drivers of this activation remain incompletely understood but are thought to include ongoing HIV replication [18, 19], secondary coinfections [20, 21], and HIV-mediated breakdown of the intestinal mucosa and subsequent exposure to gut microbial products [22]. However, strategies targeting these root drivers of inflammation such as ART intensification [23–25], treatment of coinfections [26, 27], and agents that promote mucosal repair in the gut-associated lymphoid tissue (GALT) [28, 29] are unable to completely resolve this persistent immune activation and inflammation.

Although new antiretroviral (ARV) drugs are less toxic and are associated with fewer metabolic complications, metabolic abnormalities persist in HIV patients on ART (reviewed in [30]). Factors driving these abnormalities include not only the effects of the drugs themselves but also the effects of chronic inflammation, the irreversible damage of metabolic tissues sustained prior to the introduction of ART, host genetic risk, side effects associated with other medications, age-related factors, obesity and lifestyle/behaviour (diet, exercise, and smoking) [31, 32]. Emerging evidence suggests that these metabolic abnormalities may further affect immune function and contribute to the development of non-AIDS-associated comorbidities [33, 34]. Consistent with these findings, immunometabolic signatures that combine markers of immune activation/inflammation and metabolite profiles have been shown to be strong predictors of frailty [35], hepatic dysfunction [36], neurocognitive impairment [37], and depression [38] in HIV patients on ART. However, the molecular mechanisms underlying these relationships remain incompletely characterized.

Immune responses are highly dependent on the metabolic microenvironment, which alters the cell's metabolic status and induces effector function. This metabolic reprogramming is required to meet the bioenergetic and biosynthetic demands of the cell and to activate and regulate gene expression, signal transduction, and epigenetic profiles [39, 40]. By altering cellular metabolism, it may be possible to shape and fine tune innate and adaptive immune responses [39]. Conversely, disruption of these interactions has been shown to underlie the development of many noncommunicable diseases such as CVD and type 2 diabetes [40]. In this review, we will discuss the range of metabolic abnormalities observed in HIV patients on ART and explore emerging evidence that suggests that these metabolic abnormalities may play a critical role in both supporting and driving chronic immune activation and inflammation in HIV infection.

### 1.1. Spectrum of Metabolic Abnormalities in HIV Patients on ART

Despite the successes of ART in reducing AIDS-associated morbidity and mortality, HIV-infected patient populations are experiencing decreased metabolic control and increased rates of metabolic diseases [30, 31]. Many of these diseases are associated with dysregulated lipid and glucose metabolism including dyslipidemia, insulin resistance and type 2 diabetes, CVD, and nonalcoholic fatty liver disease (NAFLD).

#### 1.1.1. Dyslipidemia

Dyslipidemia and altered fat distribution (loss of subcutaneous fat and a relative increase in central fat) are commonly observed in HIV patients on ART [41, 42]. The prevalence of these disturbances varies widely and depends on the cohort, the fat type, and the anatomic location of the adipose tissue [42, 43]. The type and duration of ART have also been shown to differentially alter lipid metabolism. The most pronounced effects are commonly observed with protease inhibitors (PI), which increase central obesity and lipoatrophy as well as alter circulating triglycerides, low-density lipoprotein cholesterol (LDL-C), and high-density lipoprotein cholesterol (HDL-C) levels [44, 45]. These alterations are associated with the inhibition of lipogenesis, adipocyte differentiation, a decrease in hepatocyte clearance of chylomicron and very low-density lipoproteins (VLDL), and the stimulation of hepatic triglyceride synthesis [45]. Certain nucleoside reverse transcriptase inhibitors (NRTI) and non-NRTIs (NNRTI) also alter adipogenesis and adipocyte differentiation [43]. Some NRTIs are also associated with mitochondrial dysfunction, which has been shown to induce adipocyte cell death and contribute to lipodystrophy [46–49]. In addition to the effects of ART, persistent inflammation in treated HIV patients has also been shown to modify lipid profiles by altering lipid processing and transport and enhancing oxidative and enzymatic modifications of lipid molecules (reviewed in [42]). Importantly, lipids are potent immunomodulatory molecules that can exert inflammatory or anti-inflammatory effects locally and systemically [50]. In HIV patients on ART, alterations in lipid profiles may feed forward and drive further immune activation and inflammation [51].

#### 1.1.2. Insulin Resistance and Type 2 Diabetes

Insulin resistance and type 2 diabetes are also common in HIV-infected patients receiving ART [52, 53]. In the Multicenter AIDS Cohort Study, the incidence of type 2 diabetes was found to be more than four times greater in HIV-infected men receiving HAART than in HIV-seronegative control participants [52]. Complex interrelationships between genetic predisposition, disease-related body changes, and metabolic effects of ART drugs have been shown to contribute to altered glucose homeostasis [54]. Protease inhibitors have been shown to directly target glucose transporter 4 (GLUT4) expression, a glucose transporter expressed by adipocytes and skeletal muscle cells. This decreased expression was found to impair insulin-mediated uptake of glucose into adipocytes [54–56]. PI-induced alterations in glucose transport and/or metabolism have also been shown to contribute to impaired insulin secretion from *β*-cells [57] and altered glycogen synthesis by hepatocytes [58], which may increase the risk of developing type 2 diabetes [59]. On the other hand, NRTIs have been shown to affect glucose metabolism and insulin resistance by inducing mitochondrial dysfunction [60–62]. Insulin resistance may also develop through indirect effects of regional body adipose tissue changes, inflammation, adipokine production, and free fatty acid dysregulation on glucose homeostasis [63–66]. Importantly, increased levels of circulating glucose may support chronic immune activation and inflammation in HIV patients on ART [67].

#### 1.1.3. Cardiovascular Disease

CVD is now the leading cause of morbidity in ART-treated patients and accounts for >10% of premature deaths [2]. It has been reported that HIV-infected individuals on ART have a twofold higher risk of CVD compared to matched uninfected individuals [68] and that 41.3% of ART-treated patients are at high risk for CVD [69]. Even those with low Framingham risk scores and no symptoms of CVD have a higher incidence of coronary atherosclerosis [70–72] and an increased prevalence of inflamed and noncalcified atherosclerotic plaques that are vulnerable to rupture [70, 73]. This increase in atherosclerosis is associated with a twofold higher risk of myocardial infarction and fourfold higher risk of sudden cardiac death [74, 75]. Large observational studies have shown that certain ART drugs/regimens are associated with an increased CVD risk [76–78], which is thought to be associated with their metabolic side effects such as dyslipidemia [77], glucose intolerance [52], and lipodystrophy [79, 80]. Inflammation is also a key factor driving the development of HIV-associated CVD. Inflammatory markers, including high-sensitivity C-reactive protein (hsCRP), interleukin 6 (IL-6), and D-dimer, are strong predictors of cardiac disease and mortality in HIV-infected patients with known CVD [72, 81, 82]. Recent studies also suggest that markers of monocyte activation (soluble and cell associated) may represent powerful predictors of early or subclinical CVD [73, 83]. Soluble CD163 (sCD163) and soluble CD14 (sCD14) levels were found to be positively associated with the formation of noncalcified or vulnerable plaques [83] and with arterial inflammation as assessed by fluorine-2-deoxy-D-glucose positron emission tomography (18FDG-PET) in HIV patients on ART [73]. Interestingly, a recent study also found that increased expression of GLUT1 on monocytes is associated with markers of CVD risk in HIV-infected individuals suggesting that increased glucose uptake may be a more specific marker of monocyte activation [84]. Additional studies are required to systematically examine relationships between altered metabolic abnormalities and persistent inflammation in HIV patients on ART with CVD.

#### 1.1.4. Nonalcoholic Fatty Liver Disease

Liver-related diseases are becoming increasingly prominent in HIV-infected patients [85, 86]. Coinfection with Hepatitis B virus (HBV) and Hepatitis C virus (HCV) significantly contribute to this burden and increase the risk for liver-related mortality [87, 88]. However, even in the absence of HBV and HCV, monoinfected individuals have been shown to experience increased rates of liver disease including NAFLD [89–91]. This increase in NAFLD is traditionally thought to be associated with ART-related hepatotoxicity as well as ART-related lipodystrophy, dyslipidemia, and insulin resistance [85]. However, emerging studies suggest that ongoing inflammation and persistent expression of viral proteins may also contribute to the complex etiology of this condition [92–94]. For example, Vpr has been shown to accelerate fatty acid flux to the liver, increase hepatic DNL, diminish hepatic fatty acid oxidation, and blunt hepatic VLDL-TG export via activation and signaling through GR, LXR-*α*, and PPAR*α*/*γ* [95]. Importantly, the liver is one of the primary sites of energy storage and is a central organ in glucose and lipid metabolism. Alterations in liver function may have significant effects on systemic metabolism.

### 1.2. Amplifying Effects of Obesity and Aging in HIV Patients on ART

#### 1.2.1. Role of Obesity

The prevalence of obesity is increasing among HIV-infected persons. In some cohorts, greater than 50% of infected individuals are classified as overweight or obese [96, 97]. Obesity in HIV-infected populations leads to the onset of further metabolic abnormalities and increased risk for type 2 diabetes, atherosclerosis, dyslipidemia, hypertension, and malignancies [96]. While the etiology of this morbidity is likely multifactorial, the synergistic effects of obesity on persistent systemic inflammation observed in HIV patients on ART may play a role in amplifying this risk [98, 99]. Interestingly, a recent study in mice found that ART treatment on a high-fat diet was associated with increased fat mass accumulation, impaired glucose tolerance, and potentiated insulin resistance compared to those on low-fat diet, suggesting that high-fat diets may further amplify the metabolic effects of ART [100]. This cumulative metabolic dysfunction was associated with augmented proinflammatory signaling within adipose tissue consistent with macrophage infiltration [100]. These findings need to be validated in HIV patients on ART.

#### 1.2.2. Role of Aging

By 2030, it is estimated that three out of every four HIV patients will be over the age of 50, more than 80% will have at least one age-related disease and approximately one-third will have at least three age-related diseases [101]. In uninfected populations, aging is associated with alterations in body composition [102, 103], a decrease in the resting metabolic rate (RMR) and variability in oxygen consumption and free radical production leading to increased oxidative stress [104–108]. Further, aging is associated with dysregulation of the immune system via immune exhaustion (immunosenescence) and chronic inflammation (inflammaging) [109, 110]. The impact of aging on metabolic and inflammatory dysregulation in ART-treated patients is not fully understood but it is expected to further increase the rates of non-AIDS-associated comorbidities in these populations. Interestingly, it has been hypothesized that age-associated alterations in cellular metabolism and reduced clearance of metabolic waste may represent a major source of inflammatory stimuli driving age-associated chronic inflammation, termed inflammaging [111]. While further studies are required to confirm these findings, preliminary studies may support this hypothesis in HIV patients on ART [37].

### 1.3. Alterations in the Metabolic Microenvironment in HIV Patients on ART

To date, metabolic abnormalities in HIV patients on ART have been primarily described using established clinical markers of metabolic function such as total cholesterol, HDL-C, LDL-C, triglycerides, HOMA-IR, and panels to assess kidney and liver function. However, these biomarkers represent only a subset of the total metabolic processes that may be altered in HIV infection. Systems-based approaches such as metabolomics have started to provide critical new insights into the spectrum of metabolic abnormalities observed in HIV patients on ART.

#### 1.3.1. Altered Plasma/Serum Metabolome in HIV Patients on ART

Plasma/serum is the primary carrier of >4000 small molecules in the body and plays a critical role in transporting nutrients and metabolic waste to the various organ systems [112]. Using metabolomic approaches to characterize abnormalities, a recent study found that good immunological response (>250 CD4^+^ cells/*μ*l after 36 months on ART) was associated with increased HDL, total cholesterol, branched chain amino acids, and tyrosine levels where as poor immunological response was associated with increased levels of glycolytic metabolites, LDL, and VLDL [113]. Another study identified five different metabolite ratios capable of accurately identifying rapid progressors or immunological nonresponders on ART at baseline. These ratios used metabolites associated with acylcarnitine hydroxylation and carboxylation as well as the catabolism of branched chain amino acids, lysine, organic acids, and tryptophan [114]. Untargeted approaches have also identified unique signatures associated with PI vs. NNRTI-based treatment regimens. Consistent with previous studies, PI treatment was shown to have significant effects on the plasma lipid profiles including altered bile acids, sulfated steroid, polyunsaturated fatty acid, and eicosanoid metabolism [36]. These alterations had substantial overlap with those reported in NAFLD and were found to correlate with markers of inflammation, microbial translocation, and hepatic function [36]. Alternatively, nuclear magnetic resonance (NMR) spectroscopy analysis found that VLDL metabolism, total triglyceride levels, and lactate levels were independently associated with the use of NNRTI-based ART [113]. The authors also suggested that these metabolite profiles were better predictors of HIV/ART-related dyslipidemia and atherogenic risk than conventional lipid measurements. Interestingly, several metabolomic studies have identified mitochondrial metabolism as an important player in the development of non-AIDS-associated comorbidities. Bailin et al. [115] found that insulin resistance in HIV patients on ART is associated with lower plasma levels of acylcarnitines and decreased acylcarnitine : FFA ratios suggesting that impaired fatty acid uptake and/or mitochondrial oxidation is a central aspect of glucose intolerance in this population. Alternatively, Okeke et al. [116] suggested that short-chain dicarboxylacyl carnitines as well as glutamine and valine metabolites may represent valuable markers of CVD risk in HIV patients on ART. The potential mechanisms driving mitochondrial dysfunction are described below.

#### 1.3.2. Metabolite Profiles in Other Biological Fluids from HIV Patients on ART

In addition to the alterations in plasma/serum metabolome, studies have identified altered metabolite profiles in a variety of other biological fluids. In cerebrospinal fluid, alterations in aspartate and glutamine metabolism, phenylalanine and tyrosine metabolism, carnitine metabolism, and energy metabolites were found to be correlated with neurocognitive impairment and increased inflammation in HIV-infected individuals on ART [37]. These alterations overlapped with metabolic alterations seen in HIV-negative older individuals suggesting accelerated aging in patients taking ART [37]. Similarly, Dickens et al. [117] found that defects in executive function, recall, and motor function were associated with increased levels of energy metabolites, such as lactate and citrate in the CSF from HIV patients on ART. These metabolites were found to differentiate groups with worsening neurocognitive function from individuals with improving neurocognitive function [117]. In oral wash samples, HIV-infected patients had elevated levels of p-cresol sulfate, nucleotides, phenylalanine, and tryptophan and decreased levels of fucose, fumarate, and N-acetylglucosamine [118]. Interestingly, a recent study found that metabolite differences in bronchoalveolar lavage fluid from HIV patients in ART were predominantly in bacterial products likely reflecting alterations in the lung microbiome [119]. Further studies are required to better characterize other interactions between these altered tissue microenvironments and immune dysfunction in HIV patients on ART.

### 1.4. Cellular Metabolic Reprogramming in HIV Patients on ART

In addition to alterations in the metabolic microenvironment, recent studies suggest that immune cells undergo dysregulated metabolic reprogramming in viral infections. Innate and adaptive immune function is intimately linked to metabolism. This reprogramming not only supports increased bioenergetic and anabolic demands but also drives specific effector functions [39, 40]. In antiviral immune responses, type I IFNs regulate protein synthesis, proliferation, membrane composition, and the nutritional microenvironment to prevent viral entry and inhibit viral replication [120–122]. A detailed review of IFN-mediated reprogramming is beyond the scope of this review and can be found elsewhere [123]. On the other hand, viruses are obligate parasites that are completely reliant on host cell metabolism. They manipulate cellular metabolism including glycolysis, fatty acid synthesis, and glutaminolysis to create an intracellular metabolic niche, which supports virion production and promotes survival of infected cells. This intracellular niche is highly specific and dependent on the virus. For example, Dengue and Zika infections increase glycolytic flux and mitochondrial ATP production to support infection [124, 125]. Alternatively, HBV and HCV depend on aerobic glycolysis to support ATP production and reprogram mitochondrial function for ROS production [126, 127]. At the level of lipid metabolism, Dengue and Vaccinia viruses use newly generated fatty acids as a means of producing ATP via fatty acid oxidation (FAO) [128, 129]. However, others hijack cholesterol metabolism and redistribute newly synthesized cholesterol to generate virion membranes [130, 131]. Given the critical role of cellular metabolism in supporting viral infections, an in-depth understanding of HIV-associated metabolic reprogramming may identify novel biomarkers and therapeutic targets to treat infection.

#### 1.4.1. Increased Cellular Bioenergetics in HIV Infection

The field is just beginning to understand the implications of metabolic reprogramming on HIV infection. Among the best characterized alterations are related to glucose metabolism. Glucose is a central source of energy and biosynthetic precursors that feed into glycolysis, pentose phosphate pathway (PPP), tricarboxylic acid (TCA) cycle, and oxidative phosphorylation (OXPHOS) [132, 133]. During HIV infection, glucose metabolism is elevated at the level of glucose transport and pathway flux [67, 134–136]. Recent studies have reported increased Glut1 expression on both monocytes and CD4^+^ T cells during HIV infection [67, 84, 137]. This upregulation on CD4^+^ T cells is associated with increased T cell activation (CD38, HLA-DR) and is inversely correlated with absolute CD4 count irrespective of treatment status [137]. Interestingly, this upregulation persists in immunological nonresponders on long-term ART even when other markers of activation have returned to almost normal levels. Consistent with these findings, Masson et al. [138] identified a polymorphism within the GLUT1 gene SLC2A1, which elevates GLUT1 expression on CD4 T cells, that was linked to poor CD4 T cell recovery and HIV disease progression in untreated and treated individuals. On monocytes, the highest levels of Glut1 expression and glucose uptake are found on proinflammatory monocyte subsets [84]. This elevated glucose transport is associated with increased levels of inflammatory cytokine production (TNF-*α* and IL-6) [34] and has been linked to subclinical cardiovascular disease [84] and frailty in HIV patients on ART [35]. Given these findings, there is an increasing interest in GLUT1 as a possible target for limiting inflammation and the development of comorbidities in HIV patients on ART. In addition to glucose, other carbohydrates, fatty acids, and amino acids serve as alternative energy sources to support ATP production. Hegedus et al. [134] found that CD4^+^ T cells cultured in galactose had increased survival rates and supported lower levels of HIV replication compared to culturing under standard conditions. Studies also suggest that alternative energy sources, such as galactose, can amplify antiviral immunity [139]. These findings may open new avenues of investigation.

#### 1.4.2. Altered Lipid Biosynthesis in HIV Infection

Lipids are a central component of the plasma membrane and other cellular compartments including the nuclear membrane, the endoplasmic reticulum, the Golgi apparatus, and trafficking vesicles such as endosomes and lysosomes. They also serve as a source of energy and facilitate signaling for a wide range of cellular processes [140, 141]. HIV has been shown to promote fatty acid and cholesterol biosynthesis to support viral replication, which requires a significant supply of free FAs, LDL, and apolipoprotein A1 [142, 143]. Recent studies suggest that Nef plays a central role in this reprogramming [144–147]. Transcriptional profiling of CD4^+^ T cells infected with wild-type vs. Nef-deleted HIV found dysregulated expression of genes associated with lipid synthesis and fatty acid oxidation, which correlated with decreased cellular lipid content [146]. This may be due to Nef-mediated suppression of PPAR*γ* expression, which transcriptionally regulates lipid metabolism, glucose homeostasis, and cellular differentiation [147]. Alternatively, Nef transfected into cell lines has been shown to increase cholesterol synthesis and transport of cholesterol to lipid rafts to facilitate viral entry and assembly [144]. Interestingly, monocytes actively increase cholesterol efflux in early stages of HIV infection. However, over time, this efflux decreases and cholesterol accumulates causing the formation of foam cells [148]. Further characterization of lipid reprogramming in both HIV-infected and HIV-uninfected bystander cells may identify novel mechanisms driving HIV pathogenesis.

#### 1.4.3. Altered Amino Acid Metabolism in HIV Infection

Dysregulated amino acid metabolism is also a central feature of metabolic reprogramming in HIV infections. Alterations in tryptophan (Trp) metabolism are among the best characterized given the immunomodulatory role of Trp and its catabolites [149, 150]. Trp is an essential amino acid that can be metabolized by tetrahydrobiopterin-dependent tryptophan-hydroxylase to produce serotonin or catabolized by TRP 2,3-dioxygenase (TDO) and indoleamine 2,3-dioxygenase (IDO-1) to produce kynurenine and its derivatives. This catabolic breakdown via the kynurenine pathway also contributes to the biosynthesis of nicotinamide adenosine dinucleotides (NAD/NADH). During HIV infection, IFN responses and TLR engagement induce intracellular IDO expression in macrophages and dendritic cells, which increase circulating levels of immunomodulatory tryptophan catabolites such as kynurenine and quinolinic acid [151]. This increase in catabolites is associated with higher levels of immune activation and inflammatory cytokine production and has been shown to correlate with the degree of microbial translocation [151, 152]. Increased IDO-1 activity has also been shown to skew CD4^+^ T cell differentiation into regulatory T cells (Tregs) instead of T helper (Th17) cells and directly impair T cell responses [151, 152]. Further, IDO inhibitor 1-methyl-tryptophan (1-MT) has been shown to restore CD4 and CD8 T cell proliferation and T cell activation to normal levels during HIV infection in vitro [153, 154]. In SIV models, 1-MT was shown to lower plasma and lymph node viral loads and increase levels of serum tryptophan [155]. Altered tryptophan metabolism has also been linked to the development of neurological disorders in HIV patients on ART. Increased flux down the kynurenine pathway not only limits serotonin production but is also associated with increased levels of kynurenine metabolites that have neurotoxic properties [156]. Preliminary studies have started to examine the role of other amino acid pathways in HIV infection. In particular, diminished breakdown of phenylalanine (Phe) to tyrosine (Tyr) has been shown to correlate with the expression of neopterin, as well as with HIV-RNA levels and CD4 counts in HIV-infected individuals [156, 157]. This decrease may be linked to reduced phenylalanine 4-hydroxylase (PAH) activity due to a loss of neopterin, a precursor of PAH's cofactor tetrahydrobiopterin [157]. Importantly, PAH and tetrahydrobiopterin play a critical role in catecholamine synthesis such a dopamine. Decreased availability of dopamine in the central nervous system has been shown to correlate with decreased neuropsychological functions, which cannot be fully rescued by ART [158].

### 1.5. Targeting mTOR and AMPK to Treat HIV Infection

Mechanistic targets of rapamycin (mTOR) and AMP-activated protein kinase (AMPK) are master regulators of metabolic gene expression, which affect cellular metabolism during viral infections [159, 160]. Emerging evidence suggests that both mTOR and AMPK may play a central role in HIV pathogenesis and may represent new targets to regulate viral replication. The mTOR complex is composed of two distinct complexes, mTORC1 and mTORC2, with distinct signaling activities. mTORC1 functions as a nutrient sensor, capable of controlling protein synthesis while mTORC2 regulates cytoskeleton organization, metabolism, and cell survival [161]. Recent studies have shown that HIV induces mTOR phosphorylation and activation and that rapamycin, a potent and specific inhibitor of mTORC1, inhibits virus replication both in vitro and in vivo [162–164]. The mechanisms underlying mTOR-mediated inhibition of viral replication are likely multifactorial including direct effects on Tat-dependent and Tat-independent transactivation of HIV promoter [165] as well as Gag- and Vif-mediated commandeering of mTOR-associated lysosome positioning and trafficking, which mediate virus trafficking, assembly, and/or budding [166]. mTOR inhibition may also limit viral replication by correcting immune dysfunction commonly observed in HIV patients such as enhanced antiviral responses and increased memory CD8^+^ T cell formation, reduced inflammatory cytokine production, and reversal of PD-1-mediated T cell exhaustion [164, 167–169]. The role of AMPK in HIV infection is less clearly defined. AMPK is a central regulator of energy expenditure and fatty acid biosynthesis, which has been shown to modulate EF2 and TSC2/mTOR pathways including NF-*κ*B activation [170]. This regulation of energy expenditure is mediated by NAD^+^ metabolism and SIRT1 activity [171]. In HIV-infected HeLa cells, activation of AMPK and SIRT1 has been shown to inhibit Tat-induced HIV LTR transcription and replication [172]. Conversely, Tat-induced reductions in NAD^+^ levels and SIRT1 activity have been shown to diminish AMPK activity [172]. Consistent with these findings, restoration of NAD^+^ levels by tanshinone II-induced nicotinamide phosphoribosyltransferase (Nampt) expression and increased AMPK activation resulted in reduced Tat-mediated HIV transactivation and reduced reactive oxygen species production [173]. Alternatively, AMPK activators such as metformin or AICAR are currently being tested with clinical trials to reactive latent HIV infection. However, emerging evidence suggests that AMPK is not directly involved in viral reactivation but may instead coordinate the process via PKC [174]. Based on these observations, more research is required to better understand the complexities surrounding AMPK's role during HIV infection.

### 1.6. Central Role of Mitochondrial Reprogramming in Driving Chronic Inflammation and Immune Dysfunction in HIV Patients on ART

Mitochondria are highly dynamic organelles that regulate a variety of cellular functions including cellular respiration and energy production, cellular metabolism, cell signaling, cell death and proliferation, Ca^2+^ homeostasis, ROS production and redox status [175]. To ensure appropriate and timely function, mitochondrial structure and morphology is tightly regulated by the processes of mitochondrial fission, fusion, and mitophagy [175]. Dysregulation of these processes leads to altered cellular metabolism, increased ROS, and increased inflammatory responses and cellular senescence, which have important roles in driving metabolic and neurological diseases [176]. In the context of HIV patients on ART, mitochondrial dysfunction is driven by both the virus and ART. While it is clear that mitochondrial dysfunction contributes to the development of non-AIDS-associated comorbidities in HIV patients on ART, the underlying mechanisms remain poorly characterized.

#### 1.6.1. HIV-Associated Reprogramming of Mitochondrial Function

As with other viruses [177], HIV has been shown to contribute to mitochondrial dysfunction and apoptosis in CD4 and CD8 T cells [178, 179]. The mechanisms underlying this dysfunction include depletion of mitochondrial DNA (mtDNA), decreased mitochondrial gene expression, reprogramming of energy production via oxidative phosphorylation, and increased ROS production [180–182]. A number of viral proteins have been shown to interact with mitochondria. Tat has been shown to reduce mitochondrial membrane potential in the mouse liver, heart, and brain tissues [183] and induce ROS production [184] and dysregulate calcium homeostasis in neurons [185]. Further, the exposure of neurons to Tat leads to decreased mitochondrial diameter and perimeter as well as fragmentation and redistribution of mitochondria within the neurons, which leads to loss of mitochondrial transportation [186]. Alternatively, Vpr has been shown to reduce mitochondrial membrane potential (MMP) leading to mitochondrial swelling and apoptosis [187]. In neurons, Vpr has also been shown to affect axonal mitochondrial transport and ATP production, which may contribute to the development of HIV-associated neurocognitive disorder (HAND) [188]. Similarly, envelope glycoprotein 120 (gp120) induces dysregulation of calcium homeostasis, increases oxidative stress, and drives apoptosis in neurons [185, 189]. Further, gp120 has been shown to increase mitochondrial fusion/mitochondrial size [190] and increase extracellular acidification rate (ECAR), which correlates with neuroinflammation and neurodegeneration primary mouse neurons in vitro [191]. Collectively, these findings suggest that HIV and associated protein may have differential effects on specific cell populations.

#### 1.6.2. ART-Induced Alterations in Mitochondrial Function and Metabolism

ARV drugs are also an important driver of mitochondrial dysfunction in HIV patient on ART [192]. While first-generation ARV drugs were associated with significant side effects in metabolically active tissues including adipose tissue, muscle tissue, and hepatic tissue, next-generation options have fewer serious adverse effects [30, 192]. Toxicity profiles of individual drugs have been examined in terms of absolute risk of metabolic complications; however, the molecular mechanisms underlying this reprogramming and the functional consequences may vary by cell type and across different tissue microenvironments.

*NRTIs* are commonly used antiretroviral drugs that represent the backbone of most first-line treatment regimens. Early studies identified pronounced mitochondrial dysfunction using NRTI given their interactions with DNA Pol-*γ*, the only known DNA polymerase in the human mitochondria [193]. Some NRTIs can act as substrates for Pol-*γ* and interfere with mtDNA synthesis, reducing mtDNA content, ETC activity, and oxidative phosphorylation and increasing mitochondrial ROS production [60, 192]. However, a number of recent studies have also reported dysfunction in the absence of mtDNA depletion suggesting secondary effects of NRTI on mitochondrial function [194]. These alterations include altered nucleotide phosphorylation and direct mitotoxic effects on mitochondrial respiration and ATP production [80, 195, 196]. In particular, AZT has been shown to inhibit the mitochondrial adenylate kinase and adenosine nucleotide translocator in isolated mitochondria [46] and promote mitochondrial ROS production by directly inhibiting the electron transport chain [196, 197]. Other NRTIs have been shown to affect complex IV activity and inhibit complex I function [60, 196, 198]. Importantly, these toxicities can be difficult to reverse and can be life-threatening and careful evaluation of the mechanisms underlying this mitochondrial dysfunction will be critical in reducing this toxicity.

*NNRTIs* do not inhibit Pol-*γ* or reduce mtDNA, but have been shown to alter mitochondrial function [192]. In particular, efavirenz (EFV) has been shown to increase mitochondrial mass, decrease mitochondrial membrane potential, increase ROS production, and induce apoptosis via intrinsic pathway (cytochrome c release and caspase 9 activity) in hepatic cell lines [199]. Blas-García et al. [195] also found that EFV inhibits complex I leading to increased ROS production and decrease ATP production, which drive drug-associated hepatotoxicity. In neuronal cell lines and primary neurons, EFV was shown to cause mitophagy, alter mitochondrial morphology, increase mitochondrial depolarization, and decrease ATP production [200]. In mouse neurons, EFV and nevarapine (NVP) inhibit complex IV of the ETC [201]. Interestingly, Weib et al. found that these effects were worsened by coadministration of the protease inhibitor (PI) and nelfinavir (NFV).

Mitochondrial dysfunction has also been reported following *PI* treatment. In human preadipocytes, mitochondrial membrane potential was significantly decreased by saquinavir (SQV) [202]. In neuroblastoma cell lines, ritonavir (RTV) was associated with mitochondrial damage, ROS production, and apoptosis [203]. In PBMCs, however, there have been contradictory results with increased MMP and cell survival, suggesting that these effects may be dependent on the cell type and the state of cellular activation [204].

#### 1.6.3. Potential Consequences of Mitochondrial Dysfunction in HIV Patients on ART

Mitochondrial structure and function play a critical role in regulating immune cell function [205, 206]. In particular, mitochondria have been shown to act as a scaffold to facilitate the activation of immune signaling pathways including antiviral immune responses. They have also been shown to produce chemical messengers (ROS, bioactive metabolites) that activate a variety of immune functions and pathways [207–209]. In particular, mitochondrial-derived molecules, such as N-formyl peptides and mtDNA, have been shown to trigger damage-associated molecular pattern (DAMP) inflammatory responses [207]. Further, TCA cycle metabolites such as succinate and citrate have been shown to activate inflammatory responses. Specifically, succinate accumulation has been shown to stabilize HIF-1*α* and upregulate proinflammatory cytokine production (IL-1*β*) during bacterial responses [209]. Increased citrate and mitochondrial citrate carrier levels also activate inflammatory responses but instead through NF-*κ*B signaling [209]. We cannot forget that mitochondria also represent an important source of ROS, which has been shown to activate the NLRP3 inflammasome and stabilize HIF-1*α* expression and activation of NF-*κ*B [210]. Further studies are required to evaluate if any of these mechanisms contribute to persistent inflammation and chronic immune activation in HIV patient on ART.

## 2. Conclusions

Low levels of chronic inflammation and immune activation play a central role in driving the development of non-AIDS-associated comorbidities in HIV patients on ART. While the newest generation of antiretroviral drugs is associated with reduce toxicity, metabolic abnormalities persist in those on treatment [30]. Emerging evidence suggests that these metabolic abnormalities interact with immune cells to drive immune dysfunction and persistent inflammation [33, 34]. Future studies should focus on unraveling these complex interrelationships to identify new therapeutic targets that limit inflammation and improve outcomes in HIV patients on ART.
